# Application of the Intervention Mapping Framework to Develop an Integrated Twenty-first Century Core Curriculum—Part Two: Translation of MPH Core Competencies into an Integrated Theory-Based Core Curriculum

**DOI:** 10.3389/fpubh.2017.00286

**Published:** 2017-11-01

**Authors:** Jaime A. Corvin, Rita DeBate, Kate Wolfe-Quintero, Donna J. Petersen

**Affiliations:** ^1^Department of Global Health, College of Public Health, University of South Florida, Tampa, FL, United States; ^2^Department of Health Policy and Management, College of Public Health, University of South Florida, Tampa, FL, United States; ^3^USF Health, College of Public Health, University of South Florida, Tampa, FL, United States

**Keywords:** public health, MPH foundational core, competencies, experiential learning, pedagogy

## Abstract

In the twenty-first century, the dynamics of health and health care are changing, necessitating a commitment to revising traditional public health curricula to better meet present day challenges. This article describes how the College of Public Health at the University of South Florida utilized the Intervention Mapping framework to translate revised core competencies into an integrated, theory-driven core curriculum to meet the training needs of the twenty-first century public health scholar and practitioner. This process resulted in the development of four sequenced courses: *History and Systems of Public Health* and *Population Assessment I* delivered in the first semester and *Population Assessment II* and *Translation to Practice* delivered in the second semester. While the transformation process, moving from traditional public health core content to an integrated and innovative curriculum, is a challenging and daunting task, Intervention Mapping provides the ideal framework for guiding this process. Intervention mapping walks the curriculum developers from the broad goals and objectives to the finite details of a lesson plan. Throughout this process, critical lessons were learned, including the importance of being open to new ideologies and frameworks and the critical need to involve key-stakeholders in every step of the decision-making process to ensure the sustainability of the resulting integrated and theory-based curriculum. Ultimately, as a stronger curriculum emerged, the developers and instructors themselves were changed, fostering a stronger public health workforce from within.

This article is *Part 2* of a series of 3 articles published in *Frontiers of Public Health*. Other articles include:

Part 1: Application of the Intervention Mapping Framework to Develop an Integrated Twenty-first Century Core Curriculum: Mobilizing the Community to Revise the MPH Core Competencies (1).
Part 3: Application of the Intervention Mapping Framework to Develop an Integrated Twenty-first Century Core Curriculum: Curriculum Implementation and Evaluation (2).

## Background and Rationale

In the twenty-first century, the dynamics of health and health care are changing dramatically. We live in a world where politics, globalization, urbanization, and physical and social environmental factors influence health in unprecedented ways. Chronic diseases, once considered diseases of affluence, are increasingly affecting low- and middle-income countries, who now bear the greatest burden of these diseases (3). Yet, as the landscape of public health changes, health systems globally have failed to keep up with changing health concerns. Based on these concerns, and informed by recent reports from the Institute of Medicine and the Association of Schools and Programs of Public Health which called for the revision of public health curricula to better meet twenty-first century challenges, the University of South Florida’s (USF) College of Public Health (COPH) committed to revising the traditional MPH core curriculum into an integrated, theory-based, and practice based curriculum that includes content and skills to meet evolving needs (4–7). Most importantly, we committed to training a better, stronger, public health workforce, prepared to tackle these problems.

**Figure 1:**
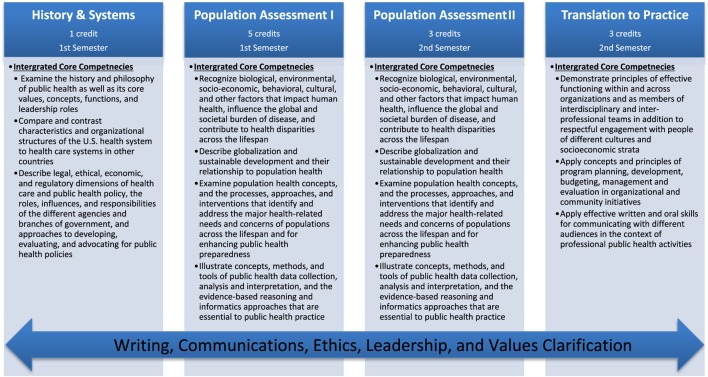
Transformed MPH course structure and associated competencies.

Driven by a multidisciplinary committee of faculty, staff, and students, an Intervention Mapping framework (8) was employed to guide the process of development, implementation, and evaluation (8). The first phase involved mobilizing faculty and increasing readiness to change the traditional five-course core curriculum (e.g., Biostatistics, Epidemiology, Health Policy and Management, Environmental Health, and Social and Behavioral Science). Once mobilized, an *ad hoc* Transforming the MPH (TMPH) committee was established to assess needs and capacity, in addition to developing revised core competencies. To guide this process, a variety of methods and processes, including town hall meetings, small group discussions, modified pile-sorting and Delphi methods were utilized. The resulting product included a revised set of twenty-first century core competencies, as outlined in Figure 1. A detailed description of the first phase of this process and the development of these competencies can be found elsewhere (blinded for review, 2017).

Holding true to the Intervention Mapping framework, once competencies were developed, the next phase focused on methods and processes used to translate the revised MPH core competencies into an integrated, theory-based, practice based curriculum that allowed for varying stages of faculty readiness for curricular change, with the goal of better preparing our workforce (8). In addition, as the College also awards the BSPH and DrPH degrees, a critical task was to ensure clear distinctions between the revised MPH and these programs (5, 9). As the field continues to advance and public health programs proliferate, clearly distinguishing these programs is essential and, thus, our process of translating the MPH core competencies into a structured curriculum was mindful of these evolving degree dynamics. Moreover, in line with twenty-first century challenges, and our changing global environment, this program was designed to be inclusive of global health perspectives and content. Specifically, this article describes how the COPH at the USF utilized the Intervention Mapping framework to translate revised core competencies into an integrated, theory-driven core curriculum that meets the training needs of the twenty-first century public health scholar and practitioner.

## Competencies and Standards Underlying the Activity

As a competency-based program, the core curriculum at the USF COPH was originally designed and developed in accordance with the core competencies and accreditation standards set forth by the Council on Education for Public Health (CEPH). However, as the TMPH committee began their task of transforming the curriculum at USF, our accrediting body was also in the process of revising and adopting a new set of core competencies. Thus, the TMPH committee had to be mindful of both the current set of standards and the proposed standards. To ensure that the committee was well informed of the ongoing CEPH revisions, members of the TMPH committee attended all sessions hosted by CEPH or ASPPH focused on the proposed revisions and participated in CEPH workgroup sessions. This helped with the daunting task of ensuring that the transformation of the curriculum at USF would align with the of the revised 2016 accreditation criteria. The resulting curriculum, discussed in this article, ensures that graduates of the program are grounded in foundational public health knowledge with a focus on the profession and science of public health and the factors related to human health. The program also meets CEPH’s 22 MPH foundational competencies, which were informed by the traditional public health core knowledge areas (biostatistics, environmental health, epidemiology, health services administration, social and behavioral sciences), and are built around: (a) evidence-based approaches to public health, (b) public health and health-care systems, (c) planning and management to promote health, (d) policy in public health, (e) leadership, (f) communication, (g) interprofessional practice, and (h) systems thinking.

## The Learning Environment

The COPH at the USF was founded in statute by the Florida Legislature in 1984 as the first school of public health in the State of Florida. The COPH is fully accredited by the CEPH and has awarded 1,576 undergraduate, 4,235 masters, and 268 doctoral degrees through the Fall of 2016. In the Spring of 2016, 1,563 students were enrolled as degree seeking students at the COPH (*n* = 729 undergraduate, *n* = 834 graduate). Of those students, 72% were women, 46% were underrepresented minorities, 76% were Florida residents, and 10% were international students. The College is also home to 293 faculty and 121 professional and support staff.

To meet the charge to transform the curriculum and ensure representation of the entire COPH community, a review committee comprised of administrative leaders, faculty from each department, instructors of current core courses, educational support staff, and students was appointed to serve as the TMPH committee.

## Employing Intervention Mapping to Guide a Revised Pedagogical Framework Overview

**Figure 2:**
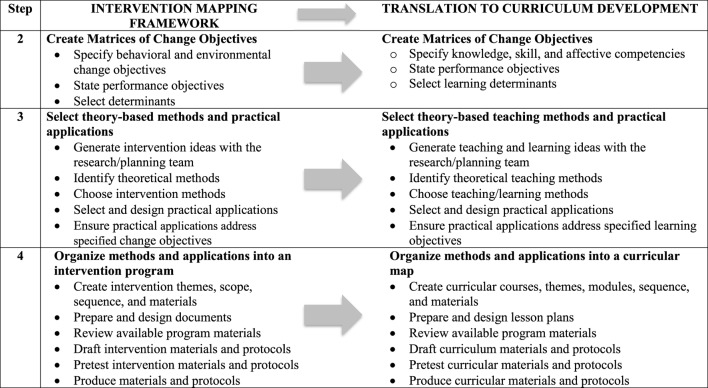
Intervention Mapping framework translating Steps 2–4 applied to curriculum development.

The Intervention Mapping framework was employed to guide the development of an integrated MPH core curriculum that addresses the revised core competencies. The process for revising these competencies is outlined in the first article of this series (blinded for review, 2017). Intervention Mapping is a systematic process comprised of six steps that facilitate the translation of competencies to teaching and learning strategies in addition to guiding theory-based decision-making with regard to curriculum mapping and developing specific lesson plans (8). In this article, we focus on the process and outcomes of Intervention Mapping Steps 2–4. As described in Figure 2, Step 2 focuses on the creation of matrices that translate competencies into specific performance objectives and learning determinants. Step 3 enables the selection of theories and methods that will be applied within the curriculum to meet learning determinants, performance objectives, and ultimately competencies. These steps provide a foundation for Step 4, which is the development of a curricular map that establishes the courses, modules, and lessons to be delivered in the new program.

### Intervention Mapping Step 2: Developing Matrices of Change Objectives

Step 2 of the Intervention Mapping process focuses on creating matrices of change objectives based on established competencies. This includes specifying knowledge, skill, and affective *performance objectives* (i.e., what do you want them to know? be able to perform? value?). It also includes identifying critical *learning determinants* for each performance objective (i.e., what is the learning determinant that will enable each specific performance objective?).

Before tackling each task in Step 2, the TMPH curriculum committee reviewed Bloom’s Revised Taxonomy of Learning which provided the committee with a pedagogical framework for organizing skills from the most basic or benchmark (i.e., remembering and understanding), to higher level or milestone (i.e., applying and analyzing), and to the most complex or capstone (i.e., evaluating and creating) (10). The committee then proceeded to brainstorm all content areas and specific determinants by completing a matrix of change for each of the revised MPH core competencies. To streamline the process, the committee brainstormed all content areas that must be addressed for the competency to be realized. MPH core competencies and associated performance objectives are listed in Table 1.

**Table 1:** Example of Intervention Mapping Step 2 with MPH integrated core curriculum.

Competency	Performance outcomes	Learning determinants
Recognize biological, behavioral, environmental, socioeconomic, cultural, and other factors that impact human health, influence the global and societal burden of disease, and contribute to health disparities across the lifespan	Systems thinking	Characteristics of a system System theory Determinants of health status Measurement of system changes Effects of globalization

	Public health biology	Host susceptibility, genetic factors, and immunological response Biological and molecular basis for PH Genetics/genomics Agent virulence, pathogenesis, tx resistance Modes of disease transmission

	Social and behavioral determinants	Behavioral epidemiology Social epidemiology

	Concepts, models, theories	Socioecological model Levels of intervention HBM, TPB, TTM, SCT, and DOI

	Environmental health	Air, water, and food quality Food security Built environment Occupational health Solid waste

	Diversity and culture	Health disparities Cultural competence

	National and international policy	US Public Health System Global public health systems

As part of this step-wise process, after agreeing upon the performance objectives and critical content associated with each core competency, the committee then turned their focus to each individual content area to determine the learning determinants necessary to achieve each content area in enough breadth and depth to meet the competency. Subsequently, learning determinants were elaborated into learning objectives using Bloom’s Taxonomy for benchmark and milestone learning objectives, which would ultimately guide lesson plan development. This activity was done for each competency and continued until agreement was reached among the committee.

Table 2 provides an example of the Intervention Mapping process using Core Competence #4: *Recognizing biological, behavioral, environmental, socio-economic, cultural, and other factors that impact human health, influence the global and societal burden of disease, and contribute to health disparities across the lifespan*. This competency was developed in Step 1 (1). Step 2 focused on determining the public health content (performance outcomes) that is necessary for achieving this competency. In this example, the committee determined that critical areas such as systems thinking, public health biology, social and behavioral determinants, theories, environmental health, diversity/culture, and national and international policy were all needed. Once these performance outcomes were determined, the committee focused on each outcome, one-by-one, to determine the subsequent learning determinant and critical content. In this example, systems thinking is one performance outcome needed to realize the competency. To ensure breadth and depth of understanding of systems thinking, the committee determined, through debate and discussion, that students would need to understand characteristics of a system, systems theory, determinants of health status, measurement of systems changes, and effects of globalization. For each of these associated areas, the learning determinant was translated into a learning objective using appropriate benchmark and milestone objectives. For example, “characteristics of a system” was translated into “understand the characteristics of a system.” “systems theory” became “describe a systems framework (i.e., life course perspective) that integrates the life course and socioecological model for assessing and addressing public health issues.” This same process was conducted for each competency, performance outcome, and learning determinant. This process provided the vital foundation and framework that allowed the committee to then identify methods and practical approaches for delivering content.

**Table 2:** Matrix of interdisciplinary core competencies and performance objectives.

#	Interdisciplinary core competencies	Performance (content) objectives
1	Examine the history and philosophy of public health as well as its core values, concepts, functions, and leadership roles	Definitions of public health, health, and population health Sentinel events in public health Core functions Essential services Public health agency accreditation Core values, principles, tenets, and frameworks Leadership Ethics and professionalism

2	Compare and contrast characteristics and organizational structures of the U.S. health system to health-care systems in other countries	Government responsibility for public health in the US Global health-care systems Public health information infrastructure

3	Describe legal, ethical, economic, and regulatory dimensions of health care and public health policy, the roles, influences, and responsibilities of the different agencies and branches of government, and approaches to developing, evaluating, and advocating for public health policies	The US public health system US health policy Access, cost, and quality considerations

4	Recognize biological, environmental, socioeconomic, behavioral, cultural, and other factors that impact human health, influence the global and societal burden of disease, and contribute to health disparities across the lifespan	Systems thinking Theories, concepts, and models Social and behavioral determinants Public health biology Environmental health (air quality, water quality, food quality and security, built environment, occupational health and safety, toxicology, infectious agents, global environmental health, solid and hazardous waste) Diversity and culture

5	Describe globalization and sustainable development and their relationship to population health	Global environmental health

6	Examine population health concepts, and the processes, approaches, and interventions that identify and address the major health-related needs and concerns of populations across the lifespan and for enhancing public health preparedness	Epidemiology Intervention strategies Risk assessment

7	Illustrate concepts, methods, and tools of public health data collection, analysis and interpretation, and the evidence-based reasoning and informatics approaches that are essential to public health practice	Epidemiology Biostatistics Informatics SPSS

8	Demonstrate principles of effective functioning within and across organizations and as members of interdisciplinary and interprofessional teams in addition to respectful engagement with people of different cultures and socioeconomic strata	Management Leadership

9	Apply concepts and principles of program planning, development, budgeting, management and evaluation in organizational and community initiatives	Program planning and evaluation Social marketing Health communication Management Leadership

10	Apply effective written and oral skills for communicating with different audiences in the context of professional public health activities	Letter to the Editor Literature review Book review and presentation Expert interview and policy brief Data report and poster Intervention concept paper

### Intervention Mapping Step 3: Identification of Theoretical Methods and Practical Applications

Intervention Mapping Step 3 focuses on expanding the matrix of change objectives determined in Step 2, to include theoretical methods and practical applications (8). As such, Step 3 focuses on the following tasks: (a) identifying theoretical methods for addressing curricular learning objectives (i.e., employing Bloom’s Taxonomy to determine each benchmark, milestone, and capstone learning objectives); (b) selecting theoretical methods to be used in the integrated core curriculum; and (c) selecting and/or designing practical teaching and learning applications. Thus, the committee focused on each of the benchmark, milestone, and capstone learning objectives for each integrated MPH core competency with regard to theoretical learning determinants, in addition to associated learning methods. Before proceeding with the tasks in Step 3, the committee also agreed to adopt another set of principles informed by the philosophy of experiential learning (11). Generally speaking, experiential learning involves carefully chosen experiences supported by reflection, critical analysis, and synthesis that require the learner to pose questions, investigate, solve problems, construct meaning, and integrate previously developed knowledge (12). With experiential learning, learning involves a reciprocal interaction between the learner, the instructor, and the environment where the instructor’s primary role includes selecting experiences, supporting learners, ensuring emotional safety, facilitating the learning process, guiding reflection, and providing necessary information (11).

Since each objective was focused on individual level actions with regard to learning objectives, the committee selected the following learning theories and associated methods: (a) information processing theories (13) associated with engagement adoption of an idea (chunking, advance organizers, imagery, discussion, cues); (b) Elaboration Likelihood Model (14) for active learning (elaboration, making meaning of the information that is processed); and (c) Social Cognitive Theory (15) for facilitation and modeling (active learning, self-efficacy, modeling, reinforcement, facilitation, reciprocity of learner, instructor, and environment).

### Intervention Mapping Step 4a: Creating Curricular Courses, Themes, and Sequence

Once the change matrices, teaching theories and methods were established in Steps 2 and 3, they were then organized into a creative sequenced curriculum that includes the courses, themes, modules, program materials, and protocols. At this point in the process, the committee employed the concept mapping method to help members sort the themes and develop curriculum maps (16). Generally speaking, concept mapping is a structured process focused on a topic of interest (i.e., MPH core curriculum), involving input from multiple participants, that produces a pictorial view of the concepts and how they are interrelated (16). Input from the concept mapping process suggested that the courses be organized around broad public health professional activities, from examination of public health challenges to the theories and tools of evidence-gathering to the design of interventions and advocacy for policy and practice changes. As a result of the concept mapping exercise, the integrated core curriculum, as seen in Figure 1, was organized into four sequenced courses: *History and Systems of Public Health* (1 credit) and *Population Assessment I* (five credits) in the Fall, and *Population Assessment II* (three credits) and *Translation to Practice* (three credits) in the Spring. The courses are designed to be taught sequentially over two semesters and to serve as the basis for a rigorous MPH curriculum.

More specifically, the first course, *History and Systems of Public Health*, is a one-credit introductory course which examines the history and philosophy of public health and exposes students to the core values, functions, and concepts of public health, while engaging them in examining and reporting on current and future public health challenges. As the introductory course, this curriculum provides both a historical perspective, with critical discussion of past events, while engaging students in future public health challenges. This one-credit course is delivered over the first three weeks of the semester. Once the course concludes, students begin the Population Assessment I course with a strong understanding of public health past, present, and future.

*Population Assessment I and II* span Fall and Spring semesters, with five credits allocated to the first course, and three credits to the second, and are designed to engage students with critical public health content and teach students to apply public health skills to current public health challenges. Through these courses, students examine population health concepts and the approaches to addressing major health-related needs. Specifically, the courses teach the fundamentals of population assessment in public health in an interdisciplinary fashion. Students are exposed to the needed theories, constructs, and tools in an applied and integrated fashion, and are expected to apply them to current public health challenges found in case studies, published research, and public health datasets.

*Translation to Practice* is the final course in the series, and is three credits. This course prepares students to translate core public health concepts and principles into real-world public health practice. Students gain hands-on experience in conducting a needs assessment, developing an intervention plan, and producing a concept paper; this allows them to put into practice the public health concepts, principles, and skills they have learned in the other courses to solve real-world public health problems. Throughout each of these courses, students are challenged to think critically and apply knowledge. In addition, writing, communication, leadership, and practical applications of data analysis are embedded throughout.

With regard to curricular themes to frame critical content, the Population Assessment I and II courses were designed using a systems-based approach, modeled after Glass and McAtee (17) theoretical framework, which posits multidimensional levels (i.e., biological, intrapersonal, interpersonal microlevel, mezzo level, macrolevel, and global level factors) that govern health (17) Using an adapted version of this model as a structure, the courses were broken into nine modules consisting of: (1) an Introductory Module, (2) Population Health Outcomes, (3) Study of Disease, (4) Genetic and Biological System Level Factors, (5) Individual System Level Factors, (6) Interpersonal System Level Factors, (7) Mezzo Systems Level Factors, (8) Macro Systems Level Factors, and (9) Global Systems Level Factors. In each of these modules, students are exposed to critical public health content within the level being discussed. All information is presented in an integrated fashion, ensuring that students are receiving high quality instruction that reflects the appropriate breadth and depth of a rigorous MPH program. During each module, students are exposed to principles, theories, and constructs that cross disciplines in an applied and integrated fashion, while applying the epidemiologic and biostatistical concepts that are required to understand and address public health problems. Each course section focuses on the delivery of activities through which students are exposed to knowledge-based content and application of that knowledge. Specifically, content that was previously taught through traditional core courses (e.g., Epidemiology, Biostatistics, and Health Policy and Management) is presented. However, rather than teaching concepts in disciplinary-specific modalities, all concepts are taught in an integrated and applied fashion.

### Intervention Mapping Step 4b: Creation of Modules, Lesson Plans, Curricular Materials, and Protocols

Once the cluster of courses were developed with associated competencies and content, the TMPH curriculum development committee focused on creating modules and associated individual lesson plans for each class. As part of designing lessons for each course, the TMPH committee was committed to adopting best practices in teaching and learning and ensuring proper redundancy and the weaving of lessons across the program.

To frame this approach, the committee adopted experiential learning principles within a flipped classroom design (11). Based on the underlying tenants of experiential learning, individual class sessions were designed to engage students and allow for the application of knowledge and conceptual understanding to real-world problems, case studies, or other scenarios. To support this, activities were embedded into each session but also spanned across courses to ensure guided practice, opportunity for reflection, and individual application. As an example, students are exposed to a case study about a street child in East Africa. Within the first module, students work on the case study and discuss the various systems level health disparities responsible for the poor health outcomes. This case then guides discussion in subsequent modules, where students critically analyze factors that influence quality of life, focus on the role of public health biology and genetic components that influence health, and walk through individual, community, national, and global level factors—from the environment to social support and globalization. In addition, students participate in the development of epidemiological studies to assess these issues and apply common statistical approaches to the study of public health conditions throughout each of these levels.

A primary objective was to ensure that our students complete the program prepared to understand and assess critical public health problems, to analyze and present data, and to develop public health programs that apply prevention strategies to address real-world public health problems. Therefore, the classes also focus heavily on scholarly writing, analyzing data, and public health planning, with a series of assignments designed to enhance these skills. Clear, articulate writing and the ability to synthesize a body of knowledge are vital in public health. Therefore, in the first semester, students participate in Writing Labs and are responsible for an individual writing project, which includes all aspects of scholarly writing, culminating with students taking a deep dive into the literature in a topic of their interest. During the second semester, the students use this literature review as an entre into meeting and interviewing an expert in the field, and to further guide their understanding of policy through the writing of a policy brief around their topic.

In reviewing the literature to determine pedagogical frameworks that would align with experiential learning and support the integrated nature of the curriculum, the inverted-learning or flipped classroom (18) and problem-based learning methods (19) were identified as the best options to facilitate experiential learning principles. Generally speaking, the traditional classroom method consists of lectures inside of class, with practice exercises and problem-solving outside of class. Conversely, the inverted-learning or flipped classroom method focuses on practice exercise and problem-solving during class, with video lectures and supplemental readings done outside of class (20). Within the flipped classroom format, experiential learning can also be facilitated through problem-based learning. Problem-based learning is student-centered and requires teachers to act as facilitators, and problems form the organizing focus for self-directed learning and the development of problem-solving skills (19).

Using the modified flipped class approach, students are presented with critical content pre-class. The goal is to have students review material on their own and come to class prepared to discuss and engage with the content. We believe that pre-class instruction is so important that students are provided with a summer reading assignment, due the very first day of class. The goal of this assignment, as with all the pre-class assignments, is to present materials that encourage students to think critically about relevant public health issues topics, review basic information, and come prepared to work through real-world public health problems. Once in the classroom, students engage in active learning. Students work through case studies where they must apply the theoretical content and work in teams to address public health challenges. All case studies are derived from real-world examples and require students to think critically about the issues, to address the influence of the entire system on the issue (from biological to global system level factors), to consider ethical challenges, and to understand the associated data.

## Discussion

The change process in moving from traditional public health core content to a transformed, integrated, and innovative curriculum is a daunting task. However, Intervention Mapping provides the ideal framework for guiding this process. Intervention Mapping walks the curriculum developers from the broadest goals to the finite details for achieving those goals. The curriculum developers start with assessing need and readiness, ultimately moving into developing goals and competencies, which then inform the application, processes, and protocols. Simply put, Intervention Mapping provides a systematic framework to guide the change process, turning a challenging task into a series of practical, manageable exercises.

Perhaps most importantly, Intervention Mapping, with its attention to linking activities to competencies, provides a rigorous and systematic approach to ensuring the development of content, while also ensuring the integrity of the curriculum, the quality of instruction, and the appropriateness of implementation. As developers are forced to think through the process from competency all the way though individual learning objectives, and to map this process, gaps or redundancies in content delivery become clear. In addition, with such attention to varying levels of learning, developers must think through the individual lesson plans, while also focusing attention on the overarching program competencies. The resulting curriculum is well thought out, systematically developed, consistently reviewed, and ultimately more rigorous in design.

Throughout this change process, a number of critical lessons were learned, and this process was not without challenges. The committee had to be open to new ideas, ideologies, and frameworks. Researching various guiding ideologies, modes of content delivery, and best practices, from flipped classroom approaches to experiential and case-based learning, required a great deal of time and commitment on the part of the committee. The committee reviewed literature, brought in experts in various methodologies, and solicited outside help in finding appropriate methodologies for course development and delivery. However, as the committee learned, it is imperative that during any transformational process, one continually evaluates best practices in light of resources, constraints, values, and overall mission, while remaining mindful that the resulting program should be informed by those that will ultimately be responsible for implementing and sustaining that program. Simply, the program had to provide the right fit for both the implementers (e.g., faculty) and adopters (e.g., students). It is vital that developers listen to constituents and stay true to the College’s vision and mission. These are not one size fits all processes and thus developers must select the best fit for the structure and culture of the institution.

It is also important to note that during this process, the committee and the educators involved assessed best practices in teaching and content delivery. This required an introspective look at how one teaches to inform the development of guiding principles and ideologies. While our process was focused on new content delivery, breaking from tradition and embracing change is challenging and time consuming. Further, during this process, individuals begin to question themselves and their own, current practices. Undoubtedly, this process encouraged change and the adoption of better practices in traditional classes as well as in the revised curriculum.

To move through this process, an innovative, creative, and committed team is required. It is also important that these individuals have adequate time allocated to the initiative. The curriculum committee met for 3–4 h per week over the course of two semesters to develop the program. From the onset, these individuals had to be committed to the idea of change and willing to engage in tasks outside of their traditional assignments. Ultimately, as a stronger curriculum emerged, the developers and instructors themselves were changed, fostering a stronger public health workforce from within.

## Conclusion

As the landscape of public health continues to change, it becomes imperative that the next generation of public health professionals is educated through evidence-informed best practices. This education must be mindful of changing academic environments and technological advances, while focusing on how we address the most complex of public health challenges. Much of our focus in public health is toward multicausal factors that influence health. Thus, we must provide students with the tools needed to address these issues. This requires a systems approach and necessitates a systematically designed curriculum. The curriculum must be versatile enough to incorporate emerging problems but scripted enough to ensure foundational competencies are taught in appropriate depth and breadth. Implicit in this is the need for a better trained professional public health workforce, one prepared to identify and address the complex emerging issues facing our global community. As the issues we face globally increase in complexity, the need for cross-disciplinary, integrated training that incorporates a systems-based approach and fosters critical thinking is imperative. The future of public health depends on such efforts.

## Author Contributions

All of the authors of this article contributed in the development and design of the reported curricula, as well as in the development, writing, and editing of this manuscript.

## Conflict of Interest Statement

The authors declare that the research was conducted in the absence of any commercial or financial relationships that could be construed as a potential conflict of interest.
